# Capsaicin, a Powerful ^•^OH-Inactivating Ligand

**DOI:** 10.3390/antiox9121247

**Published:** 2020-12-08

**Authors:** Adriana Pérez-González, Mario Prejanò, Nino Russo, Tiziana Marino, Annia Galano

**Affiliations:** 1 CONACYT-Universidad Autónoma Metropolitana-Iztapalapa, San Rafael Atlixco 186, Col. Vicentina, Iztapalapa, México City 09340, Mexico; adriana_perez_3@hotmail.com; 2 Department of Chemistry and Chemical Technologies, Università della Calabria (UNICAL), Calabria, 87036 Arcavacata di Rende, Italy; mario.prejano@unical.it (M.P.); nrusso@unical.it (N.R.); 3 Departamento de Química, Universidad Autónoma Metropolitana-Iztapalapa, San Rafael Atlixco 186, Col. Vicentina, Iztapalapa, México City 09340, Mexico

**Keywords:** capsaicin, Cu-complexes, fenton reaction, natural antioxidant, oxidative stress, DFT, molecular dynamics

## Abstract

Oxidative conditions are frequently enhanced by the presence of redox metal ions. In this study, the role of capsaicin (8-methyl-N-vanillyl-6-nonenamide, CAP) in copper-induced oxidative stress was investigated using density functional theory simulations. It was found that CAP has the capability to chelate Cu(II), leading to complexes that are harder to reduce than free Cu(II). CAP fully turns off the Cu(II) reduction by Asc^−^, and slows down the reduction in this cation by O_2_^•−^. Therefore, CAP is proposed as an ^•^OH-inactivating ligand by impeding the reduction in metal ions (OIL-1), hindering the production of ^•^OH via Fenton-like reactions, at physiological pH. CAP is also predicted to be an excellent antioxidant as a scavenger of ^•^OH, yielded through Fenton-like reactions (OIL-2). The reactions between CAP-Cu(II) chelates and ^•^OH were estimated to be diffusion-limited. Thus, these chelates are capable of deactivating this dangerous radical immediately after being formed by Fenton-like reactions.

## 1. Introduction

Pepper (*Capsicum annuum* L.) is an herbaceous plant that belongs to the Solanaceae family. It is cultivated worldwide in warm-climate regions such as America, central and southern Europe, Asia and tropical and subtropical Africa [1]. The increase in the total pepper production in the last decade [1,2] shows its economical and agricultural importance. Production reached 34.5 million tonnes (MT) of fresh pepper and 3.9 MT of dry pepper in 2016 [1,2]. India (1.4 MT) and China (17.5 MT) were the largest producers of dry pepper, while Mexico (2.7 MT), Indonesia (2.0 MT) and Spain (1.1 MT) were the largest producers of fresh pepper [2]. 

**Figure 1:**
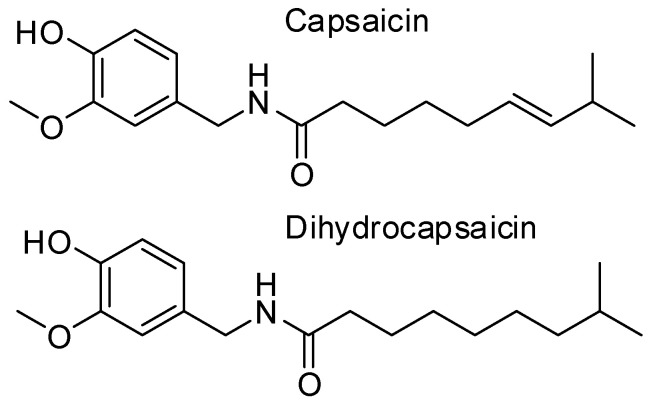
Chemical structure of capsaicinoids, bioactive compounds present in Capsicum annum varieties.

The Capsicum annum varieties contain many bioactive compounds such as flavonoids, phenolic acids, carotenoids, capsinoids and capsaicinoids (Figure 1). According to Iqbal et al. [3] capsaicin represents about 60%, and dihydrocapsaicin and about 30%, of total capsaicinoids content in pepper. The levels of capsaicinoids can vary depending on the hybrid, the climatic conditions, the maturity stage at harvest and on the storage and processing.

Some Mexican varieties of pepper contain important concentrations of capsaicinoids (1–2%). Capsaicin (CAP) and related compounds have shown diverse biological activity, which has led to envisioning them as potential candidates for pharmacological applications, for example, in the treatment of musculoskeletal and neurological pain, inflammation and oxidative-induced diseases [4]. In addition, there seems to be a relationship between the uptake of phenolic acids and the reduced risk of diabetes, coronary disorders, osteoporosis, cancer and neurodegenerative diseases [5]. There is also a general agreement of the beneficial effects that natural antioxidants may offer for preventing or delaying the occurrence of age-related cognitive deficits. Therefore, discovering specific antioxidants from natural sources has acquired new relevance, as well as identifying which of them might be used as potential therapeutic agents in the treatment of specific diseases [6,7]. 

Chili extracts (capsaicin and dihydrocapsaicin) have been studied by Ahuja, et al. [8] to analyze their effects on copper-induced oxidation of human serum lipids. It was found that the beneficial effects of capsaicin are concentration-dependent and it is involved in reducing the risk of coronary heart disease. In another study, it was reported that four weeks of regular consumption of 30 g per day of chili blend reduces the rate of copper-induced oxidation by about 10.5% in the serum of men and women [9]. Alam et al. [10] studied the importance of chili bioactive compounds present in six different varieties. The two varieties with the highest content of capsaicin and dihydrocapsaicin were identified as those exhibiting the most potent antioxidant activity.

Capsaicinoids are also reported to have other promising pharmacological activities, including protection against various strains of bacteria. The authors of [11,12,13,14] studied the structure–activity relationships of natural and synthetical capsaicinoids. They found that capsaicin and dihydrocapsaicin, are superior efflux pump inhibitors compared to the standard verapamil. Perucka et al. [15] proposed that, besides their well-known pharmacological properties, capsaicinoids are also important because of their antioxidant properties, similar to that of flavonoids. This is consistent with the previous theoretical investigation on the free-radical scavenging activity of capsaicin, proposed as a better antioxidant than melatonin and caffeine [16]. 

However, a detailed study of the potential role of capsaicin as an antioxidant in the presence of redox metals, and the chemical routes involved in such activity, has not been explored to date. Thus, this is the main goal of the present work. To that purpose, the present study focuses on the possible OIL (^•^OH-inactivating ligand) behavior of this compound [17,18]. It can be exhibited in two different ways, namely by impeding the reduction in metal ions (OIL-1) and by scavenging ^•^OH yielded through Fenton-like reactions (OIL-2) [19]. Both were explored here, considering different mechanisms and reaction sites as well as the pH of the environment. To do this, the copper metal ion, Cu(II), has been considered due to its possible role in the production of reactive oxygen species (ROS) [20], competing with iron ions, Fe(III) under the same experimental conditions to induce oxidative stress. [21,22] In fact, the ease of interconversion by oxidation and reduction reactions of copper ions, Cu(I)/Cu(II) makes copper a good candidate to promote the Fenton reaction [22,23,24,25]. Therefore, the ability of CAP to act as an antioxidant exhibiting OIL-1 and OIL-2 behavior has been tested towards Cu(II) ions to reduce the copper-induced oxidative stress. 

## 2. Computational Methods 

### 2.1. Electronic Calculations

Quantum mechanical (QM) calculations were performed using the Gaussian 09 package [26]. Geometry optimizations and frequency calculations were carried out within the framework of the Density Functional Theory (DFT), employing the M05 functional [27]. Previous studies have successfully used this functional for thermochemistry and kinetics parameters relative to reactions involving free radicals and metals [28,29,30,31,32,33,34]. The M05 functional was used in conjunction with the 6-311+G(d,p) basis set and the SMD solvation model [35] to mimic water environments. Unrestricted calculations were used for open-shell systems. For the optimized structures, their minimal nature was assured by the absence of imaginary frequencies. 

### 2.2. Molecular Dynamics

According to a well-assessed protocol [36,37], the General Amber Force Field (GAFF) [38] and the restrained electrostatic potential (RESP) method [39] were adopted to extrapolate Lennard–Jones and charges parameters on previously HF/6-31G(d)-optimized structures of the neutral and anion capsaicin. The relative parameters are provided as Supplementary Information (Table S1). 

The Molecular Dynamics simulations (MDs) were carried out in both hydrophobic and hydrophilic environments. In the first case, the behavior in the gas phase was considered, while in the second, the molecules were solvated via a box (47Å × 44Å × 42Å) of TIP3P [40] water molecules (2092). For the anion form, the cation Na^+^ was added to neutralize the negative charge. The whole systems were minimized and gradually heated from 0 to 298 K for 50 ps, and maintained to the same temperature for a further 50 ps in NVT conditions. The MDs production phase consisted of 100 ns in the NPT ensemble for both considered systems using the PME [41] and SHAKE [42] algorithms with an integration step of 0.002 fs, and considering a cutoff radius equal to 14.0 Å. All MDs were carried out using the AMBER16 software package [43]. 

### 2.3. Reduction Reactions

The quantum mechanism-based test for overall free-radical scavenging activity (QM-ORSA) protocol [28] based on the conventional transition state theory (TST) [44,45,46] the 1M standard state and the Marcus theory [47,48,49,50] were used. Calculated rate constants that were within, or close to, the diffusion-limited regime were corrected using the Collins–Kimball theory [51] in conjunction with the steady-state Smoluchowski apparent rate constant [52], and the Stokes–Einstein approaches [53,54]. More details on this protocol can be found elsewhere [28,55,56]. 

## 3. Results and Discussion

### 3.1. Conformational Analysis

**Figure 2:**
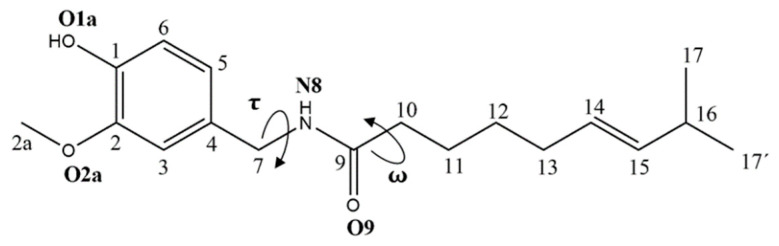
Capsaicin (CAP). Structure and chelation sites numbering.

The presence of several single rotatable bonds in the CAP chemical skeleton can make the switching from reversible open-close conformations of capsaicin possible. Therefore, a careful examination of the most important **τ** and **ω** dihedral angles was performed (Figure 2).

**Figure 3:**
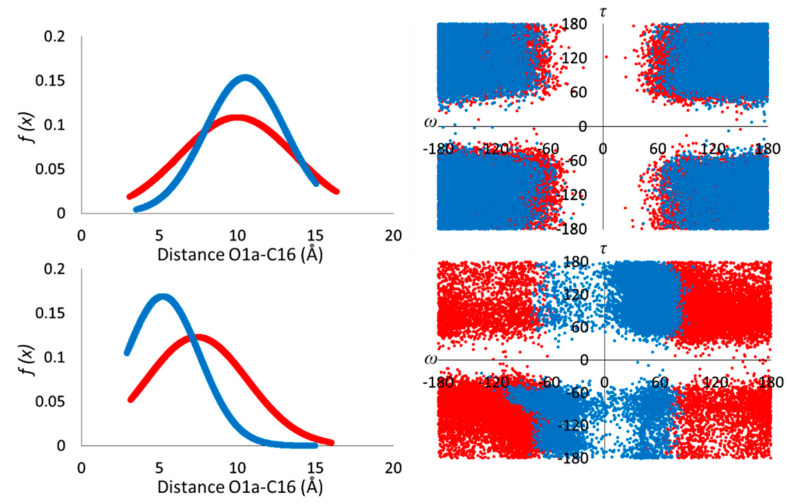
Gaussian distribution of O1a-C16 distance (left side), and Ramachandran-like plot (right side) of ω and τ dihedral angles calculated along the MDs.

The resulting RMSD trends, extrapolated by MDs, showed sensible conformational heterogeneity (Figure S1, Supporting Information), with average values of 2.39 and 2.76 Å, for neutral and anionic forms in water, and of 2.08 and 1.70 Å for neutral and anion forms in the gas phase, respectively. The Gaussian function of the head–tail distance O1a-C16 (Figure 3) in water showed a similar distribution for the two species, with functions centered at the average values of 10.04 and 10.50 Å for neutral CAP and its anion, respectively, and related standard deviation σ_neutral_ = 3.68 Å and σ_anion_ = 2.60 Å. Moreover, the O1a-C16 and the dihedral angles ω (C11-C10-C9-N8) and τ (C4-C7-N8-C9) were monitored as the main parameters of the conformational behavior of the systems.

As evidenced also from the representative clusters (Figure S1, Supporting Information), and from the dispersed displacement of ω (± 60° ± 180°) and τ (± 60° ± 180°) dihedral angles (Figure 3), both forms of capsaicin in water present displacement close to the open conformation (ω; τ = ± 180°; ±180°). The CAP molecule presents four different sites that can be involved in hydrogen bond networks as acceptors (O2a and O9) and donor–acceptor systems (O1a and N8). For this reason, the variation in intra- and intermolecular hydrogen bonds was also examined during the MDs. In the presence of explicit water molecules, the CAP anion presented a three times higher number of hydrogen bonds with the solvent respect to the neutral one, as reported in Figure S2 (Supporting Information). 

In the hydrophobic (gas phase) medium, the Gaussian trends are centered to lower average values of 7.41 Å (σ = 3.24 Å) and 5.22 Å (σ_anion_ = 2.36 Å) for the neutral and anion form, respectively. The dihedral plot (right side of Figure 3) shows fewer broad distributions of ω and τ. While in the case of neutral species, a range of ω = ± 60° ± 120° and τ = ± 60° ± 120° was expressed, in the anion, the distribution is mainly localized in the proximity of ω = 0° + 60° and τ = +60° + 180°, corresponding to a closed conformation (ω; τ = ± 70°; ±70°). 

**Figure 4:**
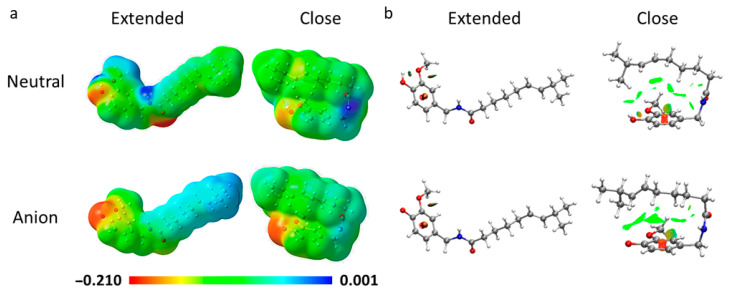
Molecular electrostatic potential (**a**) and the non-covalent interaction (NCI) plot (**b**) for both the extended and close conformations.

An interesting outcome arises from the molecular electrostatic potential for both extended and closed forms of neutral and anionic CAP (Figure 4). Independently of the acid-base form of CAP, neutral or charged, the molecular electrostatic potential (MEP) distribution is influenced by the conformation. This behavior can affect the copper chelation process since, in the folded conformation, oxygen and nitrogen atoms are accessible to the metal ion. The non-covalent interaction (NCI) plot (Figure 4) indicates the presence of weak interactions involving the olefinic bond and the aromatic ring, as evidenced by the green. 

### 3.2. Cu(II) Chelating Ability of CAP

Chelating ability is the key feature of any antioxidant exhibiting OIL behavior, regardless of the chemical route involved (OIL-1 or OIL-2). In OIL-1, the metal center in the complex is protected from reduction. Thus, the consequent ^•^OH production, via Fenton-like reactions, is inhibited. In OIL-2, the metal in the complex is reduced, i.e., it may contribute to the production of ^•^OH. Since, in this case, the ligand would be the molecular frame closer to the ^•^OH formation site, it may act as a sacrifice target, preventing this radical from reaching biomolecules such as DNA, proteins, and lipids. Therefore, the Cu(II) chelating ability of CAP was the first aspect investigated in the exploration of the potentiality of this compound as an OIL antioxidant. 

In analogy with other similar molecules that were investigated [17,18,57], and as suggested by the chemistry of CAP (that includes the monomethylated cathecol moiety), all the possible chelation sites in CAP involving N and O atoms (Figure 2) were explored, taking into account the formation of both mono-dentate (A) and bi-dentate (B) chelates. The following chemical routes were considered:

Route I: Direct chelation by neutral CAP (H_n_CAP)

Cu(H_2_O)_4_^2+^ + H_n_CAP ⇆ [Cu(H_2_O)_4−*j*_(H_n_CAP)]^2+^ + *j*H_2_O;

Route II: Direct chelation by anionic CAP (H_n−1_CAP^−^)

Cu(H_2_O)_4_^2+^ + H_n−1_CAP^−^ ⇆ [Cu(H_2_O)_4−*j*_(H_n−1_CAP)]^+^ + *j*H_2_O;

Route III: Coupled deprotonation-chelation involving neutral CAP (H_n_CAP)

Cu(H_2_O)_4_^2+^ + H_n_CAP ⇆ [Cu(H_2_O)_4−*j*_(H_n−1_CAP)]^+^ + *j*H_2_O + H^+^;

Route IV: Coupled deprotonation-chelation involving anionic CAP (H_n−1_CAP^−^)

Cu(H_2_O)_4_^2+^ + H_n−1_CAP^−^ ⇆ [Cu(H_2_O)_4−*j*_(H_n−2_CAP)] + *j*H_2_O + H^+^.

The coupled-deprotonation–chelation (CDCM) mechanism (i.e., routes III and IV) concurrently involves Cu binding and deprotonation of the chelation site in the ligand. Consequently, the associated Gibbs energy and equilibrium constant depend on the pH, which was considered here. More complete information on the calculations for these particular mechanisms, specifically on how to obtain conditional values at each pHs of interest, can be found elsewhere [57,58]. The data reported in this work correspond to physiological pH = 7.4.

Considering the known pKa of CAP, (pKa = 10.10) [59], the molar fraction of neutral and anionic forms were estimated at a physiological pH. Their relative abundance was found to be 99.8% and 0.2%, respectively, under such conditions. (Table S2 and Figure S3, Supporting Information). Despite the low amounts of the anionic form, it was taken into account in the modeling. 

To establish the energetic features of extended (CapN_ext and CapA_ext) and closed (CapN and CapA) conformations of the free ligand, the two most stable conformers extracted from MDs calculations were also minimized at the QM level. The results collected in Table 1 show that the closed arrangements have the lowest energy, although the differences are small. According to the Maxwell–Boltzmann distribution (expressed in percentage, MB%), a non-negligible population of the extended conformation is expected for both neutral and anionic CAP (11.0% and 32.3%, respectively).

The structures and acronyms of the modeled chelates are provided in Table S3 (Supporting Information). The corresponding energies, considering the different chelation sites (O1a, O2a, N8, and O9; Figure 2), are reported in Table 2. According to the data gathered in this table, among the 24 located chelates, 11 are predicted to be viable from a thermochemical point of view, i.e., they are formed through exergonic (**∆**G < 0) reaction pathways. However, only two of them (IIB-O1a-O2a and IIB-O1a-O2a_ext) are predicted to exists in significant amounts. Their populations were estimated to be 91.96% and 7.98%, respectively, based on Maxwell–Boltzmann calculations. Thus, the following investigation is focused on these two chelates.

The apparent equilibrium constants (*K^app^*) reported in Table 3 were obtained according to

(1) $$K^{app}=\sum K_{i}^{II}$$

where *K_i_^II^* represents each reaction pathway, corresponding to route IIB, that contributes to the chelation process and involves one of the two most abundant conformations of mono-anionic CAP. The *K_i_^II^* values were estimated as

(2) $$K_{i}^{II}={}^{M}f\left(H_{n-1}CAP^{-}\right)*K_{i}$$

where

(3) $$K_{i}=e^{-\Delta G_{i}^{II}/RT}$$

and ${}^{M}f\left(H_{n-1}CAP^{-}\right)$ is the fraction molar of mono-anion.

Since the estimated *K^app^* value is higher than 10^10^ M^−1^, CAP is predicted to be a strong Cu(II)-chelating agent. Therefore, the first requirement for its possible OIL behavior is fulfilled.

### 3.3. OIL-1 (Inhibiting the Reduction in Metal Ions)

As previously mentioned, OIL-1 involves sequestering metal ions from reductants. To evaluate such behavior, copper was used as the redox metal, and the superoxide radical anion (O_2_^•−^) and the ascorbate anion (Asc^−^) as the reductants. O_2_^•−^ is relevant in this context because it is known to be involved in the metal-catalyzed Haber–Weiss recombination (HWR) and it is a very strong reductant. Asc^−^, on the other hand, has a moderate strength as a reductant, but it is still able to reduce Cu(II) to Cu(I), which is a crucial step in the ^•^OH production via the Fenton reaction. In fact, Cu-ascorbate mixtures are frequently used to generate oxidative conditions in experiments, which are believed to arise as a consequence of an HWR process.

The relevant reactions were explored from both, thermochemical and kinetics, calculations. The overall rate constants were computed as

(4) $$k_{overall}=\sum k_{i}^{app}$$

In this case the contributions of each conformer (closed or extended, ${}^{MB}f\left(R_{i}\right)$) were also considered and weighted according to the Maxwell–Boltzmann distribution (Table 1). The molar fractions of the reductants (${}^{M}f\left(Red\right)$v), i.e., capN (0.998), capAn(0.002), O_2_^•−^(0.9975) and Asc^−^(0.9993) at the pH of interest (pH = 7.4) were also taken into account, to calculate *k_i_^app^*, according to

(5) $$k_{i}^{app}={}^{M}f\left(Red\right)\;{}^{MB}f\left(R_{i}\right)k_{i}^{TST}.$$

The results for all reductants are shown in Table S4, (see Supporting Information). As expected, the mono-anion species of the CAP (H_n−1_CAP^−^) are a better reductant than its neutral one.

According to the overall rate coefficient, CAP is 286.98 and 8.55 times less powerful as a reductant of free Cu(II) than O_2_^•−^ and Asc^−^, respectively. It is important to underline that the neutral species of CAP, which is most abundant at physiological *p*H, is not capable of reducing free Cu(II), since such a process is endergonic (Table S4, see Supporting Information). On the other hand, the anion can act as a Cu(II) reductant, but to a lower extent than both O_2_^•−^ and Asc^−^. These findings strongly suggest that CAP does not promote OH radical generation via Fenton-like reactions. Therefore, it is not expected to have pro-oxidant behavior in the presence of copper.

The calculated data of the reduction in Cu(II) as free copper, as well as in the CAP-Cu(II) chelates, are provided in Table 4 for the O_2_^•−^ and Asc^−^ reductants. To validate the used approach, the rate constant calculated with it was compared with the experimental value (8.1 × 10^9^ M^−1^s^−1^) [60] for the reaction between free Cu(II) and O_2_^•−^. The theoretical value was found to be 1.82 times lower than the experimental one, which constitutes an excellent agreement. Thus, the reliability of the kinetic data reported here seems to be supported.

According to the data collected in Table 4, the Gibbs free energies for the reduction reaction of Cu(II) chelates are negative with O_2_^•−^. This indicates that these processes would take place spontaneously, although in a less exergonic manner than for free Cu(II). 

The reactions involving CAP chelates are also predicted to be slower: the rate constants for the O_2_^•−^ reactions with IIB-O1a-O2a and IIB-O1a-O2a_ext complexes are 75 and 548 times lower than with Cu(II), respectively. The *k^overall^* of the CAP-Cu(II) + O_2_^•−^ reaction is 66.28 times lower than that of the Cu(II) + O_2_^•−^, at physiological pH and considering their Maxwell–Boltzmann distribution for each CAP conformer. 

Therefore, in the presence of O_2_^•−^, or similarly strong reductants, CAP may prevent copper-induced oxidation (to some extent) by inhibiting Fenton-like reactions. In other words, CAP might be moderately efficient as an OIL-1 antioxidant. On the other hand, when the reductants are not as strong as O_2_^•−^, for example, the ascorbate anion, Cu(II) chelation by CAP is expected to fully inhibit the Cu(II) reduction and the consequent ^•^OH production. The Gibbs free energies of the reactions of Asc^−^ with the CAP-Cu(II) complexes was be endergonic and the corresponding rate constants are smaller than those involving the free Cu(II) ion, indicating that CAP is predicted to prevent Cu-ascorbate-induced oxidation. 

### 3.4. OIL-2 (Scavenging ^•^OH Yielded through Fenton-Like Reactions)

**Scheme 1:**
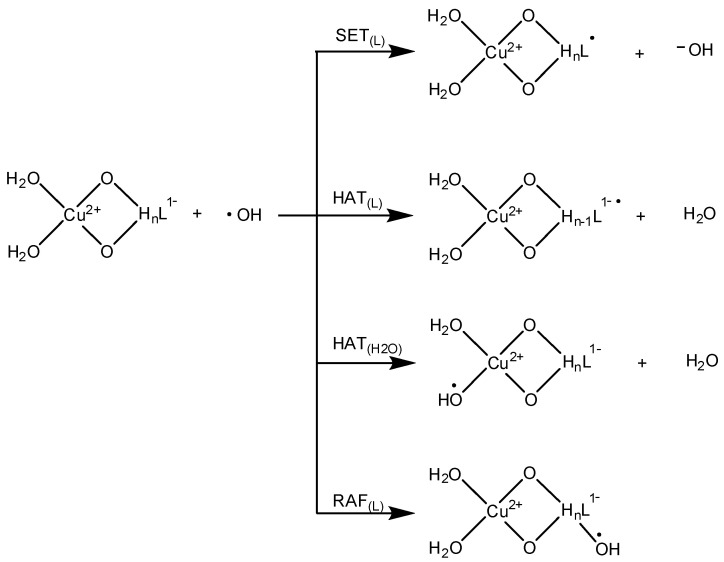
Mechanisms explored to analyze the OIL-2 activity of CAP.

The mechanisms explored to analyze the OIL-2 antioxidant behavior of CAP-Cu(II) are provided in Scheme 1. They are single-electron transfer (SET), radical adduct formation (RAF) and hydrogen atom transfer (HAT). As shown in Scheme 1, for the HAT mechanism there are different sites in which the CAP-Cu(II) chelates may act as H donors. 

These are the hydroxyl groups, when not involved in chelation, the hydrogen of the sp^3^ carbon in CAP, and the water molecules coordinated to Cu(II). The thermodynamic data for all the studied reaction pathways, at physiological pH and 298 K, are provided in Table 5.

All the reaction pathways of the different mechanisms were found to be highly exergonic and the Gibbs energies of reaction range from −42.34 to −13.16 kcal/mol, which suggests that these reactions might be very fast, and are probably diffusion-limited. This may be a consequence of the high reactivity of the OH radical. However, to test such a hypothesis, the rate constants of the SET reactions, as well as those of the most and least exergonic HAT and RAF pathways, were calculated. 

**Figure 5:**
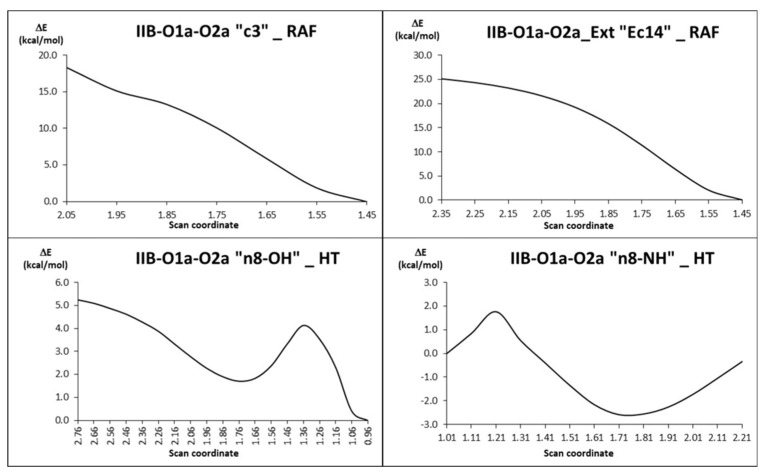
Scan coordinates of the HAT and RAF reactions between CAP-Cu(II) and ^•^OH.

For the SET mechanism, the Gibbs energies of activation are very small, i.e., 0.75 and 0.45 kcal/mol for IIB-O1a-O2a and IIB-O1a-O2a_ext, respectively. The corresponding rate constants (7.7 × 10^9^ M^−1^s^−1^) confirmed that they take place within the diffusion-limited regime. For the HAT and RAF reactions, the energy surface was explored by performing scan calculations to verify if they are barrierless or not (Figure 5). 

Concerning the RAF mechanism, the most and least exergonic paths are Ec14 and c3, respectively, where E means that this is the extended conformation of the CAP. For these two reaction sites, the scans show a curve with no maximum, meaning that they can be identified as barrierless, and their rate constants are strictly limited by diffusion and were estimated to be 9.0 × 10^9^ M^−1^s^−1^.

**Figure 6:**
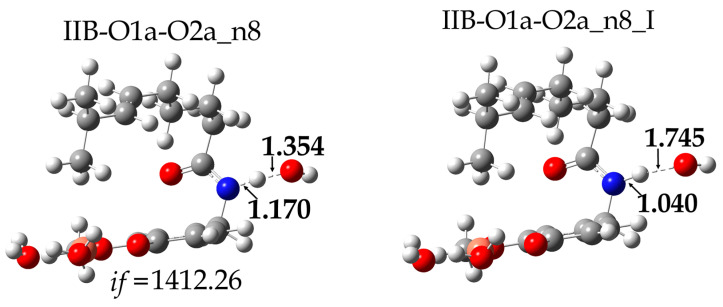
Transition state (IIB-O1a-O2a_n8) (**left**) and intermediate (IIB-O1a-O2a_n8_I) (**right**) of the reaction between CAP-Cu(II) and the OH free radical.

In the case of the HAT mechanism, the explored energy surfaces correspond to reaction sites Ec13 and n8. The scan plots of the distances C---H, O---H and N---H, associated with the bonds involved in the transfer of the H atom, were determined. For the Ec13 reaction site (the most exergonic HAT pathway) a maximum was also not found in the potential energy curves for this reaction; it is assumed that there is no energy barrier and the related rate constant is limited by diffusion. On the contrary, for the reaction path n8 (the least exergonic), a maximum was found in the potential energy curve, which corresponds to the transition state shown in Figure 6.

**Figure 7:**
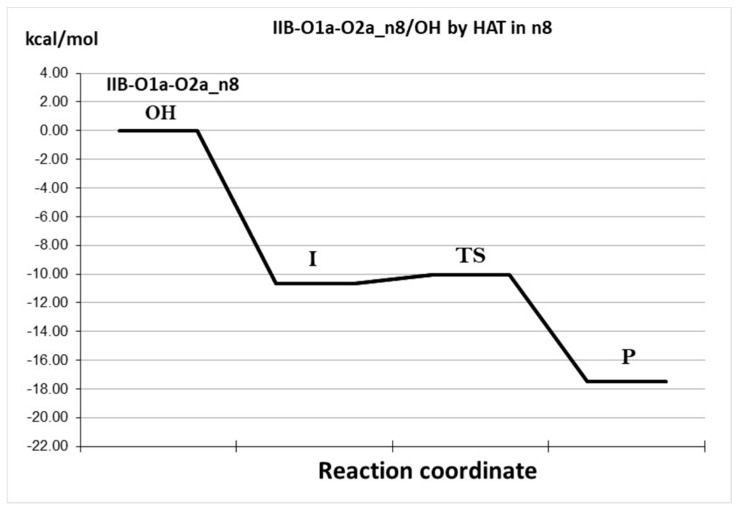
Reaction profile for the HAT reaction CAP-Cu(II) + OH, pathway n8. Isolated reactants: IR, intermediate: I, transition state: TS, products: P.

After locating this transition state, it was verified that not only does it have a single imaginary frequency, but also that it properly connects with the intended reactants and product. However, in a first attempt to estimate the associated energy of activation, it was found that the TS is 14.48 kcal/mol lower in energy than the isolated reactants. This indicates the formation of a reactant complex. Thus, it was also optimized, and its Gibbs free energy was found to be 10.67 kcal/mol lower than that of the reactants (Figure 7). Accordingly, the n8 pathway is predicted to involve two steps that correspond to a stepwise process. The first step (the formation of the reactant complex) does not involve a barrier, while the second one (the actual H transfer) has a barrier of 0.63 kcal/mol in terms of Gibbs free energy. The latter is then the rate-determining step and the calculated rate constant (9.0 × 10^9^ M^−1^s^−1^) is diffusion-controlled. 

Considering that the most and least exergonic pathways of the reactions between CAP-Cu(II) complexes and OH are controlled by diffusion, it seems reasonable to assume that this is also the case for all the other routes (with intermediate exergonicity). Considering these results, it can be stated that CAP-Cu(II) complexes are efficient scavengers of the OH free radical immediately after they are formed by Fenton-like reactions. Therefore, CAP is proposed to be an excellent OIL-2 antioxidant.

## 4. Conclusions

In this study, DFT and MD calculations were employed to predict the efficiency of capsaicin as an OIL antioxidant. 

The gathered data show that CAP can act as a Cu(II)-chelating agent that decreases the viability of Cu(II) reduction. The chelation reactions involving the CAP anion, in the extended and closed configurations, are exergonic in aqueous solution at a physiological pH. In addition, the CAP anion does not exhibit a pro-oxidant behavior in the presence of Cu(II), since it is not able to reduce this metal. It was proven to be a weaker reductant than O_2_^•−^ and Asc^−^. 

The chelation of Cu(II) by CAP inhibits the reduction in Cu(II) to Cu(I) by O_2_^•−^ 66.28 times, and by Asc^−^ 4853.91 times. However, while CAP fully turns off the Cu(II) reduction by Asc^−^, it only slows down the O_2_^•−^ reduction. Therefore, CAP is proposed as a moderate OIL-1 antioxidant. 

On the contrary, it is predicted to be an excellent OIL-2 antioxidant. The reactions of the CAP-Cu(II) chelates with ^•^OH were estimated to be diffusion-limited. Thus, they are able to deactivate this dangerous radical immediately after its being formed by Fenton-like reactions. 

## Figures and Tables

**Table 1:** Relative electronic energies (∆E, kcal/mol) for extended and closed conformation of CAP, calculated at M05/6-311+G(d,p) level of theory.

Species *	ΔE (kcal/mol)	MB%
capN	0.00	89.01
capN_ext	1.24	10.99
capA	0.00	67.66
capA_ext	0.44	32.34

* capN and capN_ext are closed and extended conformation for Neutral CAP, respectively. capA and capA_ext are closed and extended conformation for Anionic CAP, respectively.

**Table 2:** Gibbs free energies of formation (∆G°, kcal/mol) for all complexes of CAP, for neutral and anionic species, with their Maxwell-Boltzmann distribution (MB%).

Complex	∆G°	MB%
IA-O1a	3.29	-
IA-O1a_ext	5.92	-
IA-O2a	5.91	-
IA-O2a_ext	7.84	-
IA-O9	0.61	-
IA-O9_ext	2.74	-
IB-O1a-O2a	1.33	-
IB-O1a-O2a_ext	3.97	-
IIA-O1a	−17.10	0.03
IIA-O1a_ext	−17.07	0.03
IIA-O2a_ext	−5.12	-
IIA-O9_ext	2.26	-
IIB-O1a-O2a	−21.88	91.96
IIB-O1a-O2a_ext	−20.43	7.98
IIIA-O1a	−8.90	-
IIIA-O1a_ext	−8.35	-
IIIA-N8	3.25	-
IIIA-N8_ext	5.64	-
IIIB-O1a-O2a	−13.68	-
IIIB-O1a-O2a_ext	−11.71	-
IIIB-N8-O9	−3.00	-
IVA-N8	2.79	-
IVA-N8_ext	5.24	-
IVB-N8-O9	−3.00	-

**Table 3:** Apparent equilibrium constant (*K^app^*, M^−1^) corresponding to the formation of Cu(II)-CAP complexes, at 298.15 K and pH = 7.4.

Complex	ΔG°	*K_i_*	$K_{i}^{II}$
IIB-O1a-O2a	−21.88	1.09 × 10^16^	2.16 × 10^13^
IIB-O1a-O2a_ext	−20.43	9.43 × 10^14^	1.88 × 10^12^
*K^app^*			2.35 × 10^13^

**Table 4:** Gibbs free energies of reaction (ΔG°, kcal/mol), reorganization energies (λ, kcal/mol), Gibbs free energies of activation (ΔG^≠^, kcal/mol) and apparent rate coefficients (*k_i_^app^*, M^−1^s^−1^) for the reduction in the most likely Cu(II) chelates by O_2_^•^^−^ and Asc^−^. The values were calculated in aqueous solution, at 298.15 K. Free Cu(II) is included for comparison purposes.

O_2_^•−^	ΔG°	λ	ΔG^≠^	*k_i_^app^*	*k_Cu(II)_^app^*/*k_i_^app^*
			O_2_^•−^		
Cu(II)	−24.01	51.87	3.74	4.46 × 10^9^	
IIB-O1a-O2a	−13.21	50.11	6.79	5.92 × 10^7^	75.39
IIB-O1a-O2a_ext	−13.96	50.09	6.52	8.14 × 10^6^	548.32
*k^overall^*				6.73 × 10^7^	66.28
			**Asc^−^**		
Cu(II)	−4.67	34.14	6.36	1.33 × 10^8^	
IIB-O1a-O2a	6.12	32.38	11.45	2.31 × 10^4^	5744.24
IIB-O1a-O2a_ext	5.38	32.37	11.00	4.25 × 10^3^	31,316.16
*k^overall^*				2.74 × 10^4^	4853.91

**Table 5:** Gibbs free energies of reaction (ΔG°, kcal/mol), for the pathways involved in the CAP-Cu(II) chelates + ^•^OH reactions, at 298.15 K.

	IIB-O1a-O2a	IIB-O1a-O2a_ext
SET		
O1a-O2a_ct	−23.85	−24.63
HAT		
c2a	−22.35	−22.84
c7	−41.97	−41.46
n8	−17.48	−17.99
c10	−29.71	−31.44
c11	−27.34	−27.07
c12	−27.07	−27.36
c13	−39.82	−42.34
c17	−22.14	−22.18
c17p	−20.05	−22.77
o1a	−36.19	−33.99
o2a	−40.28	−38.86
RAF		
c1	−16.30	−16.51
c2	−19.19	−21.73
c3	−13.16	−13.72
c4	−17.26	−18.51
c5	−14.89	−15.32
c6	−16.40	−16.21
c14	−26.15	−26.44
c15	−26.23	−24.89

## Supplementary Materials

The following are available online at https://www.mdpi.com/2076-3921/9/12/1247/s1, Figure S1. RMSD trends calculated along molecular dynamics of neutral and anion forms of capsaicin molecule in water (top) and gas phase (bottom). On the right, the representative clusters extrapolated from the simulations. In blue, the most populated clustered geometry is represented. 2 Figure S2. Number of hydrogen bonds in the MD simulation of the neutral and anion form of the capsaicin. 3 Figure S3. Molar fractions of Capsaicin (CAP). 4 Table S1. Parameters adopted during MD simulations in AMBER package format 5 Table S2. Molar fraction of Capsaicin (8-methyl-N-vanillyl-6-nonenamide, CAP, pKa = 10.1) at different pH. 11 Table S3. Acronyms and structures of the modeled complexes. The chelation routes (CR) involved are specified, as well as the chelation sites (CS)_conformation (C) and structure. 12 Table S4. Gibbs free energies of reaction (∆G°, kcal/mol), reorganization energies ∆G, kcal/mol), Gibbs free energies of activation ∆G, kcal/mol), rate coefficients (*k_i_^app^*, M^−1^ s^−1^) and overall rate coefficients (k overall, M^−1^ s^−1^) for the reduction in the free Cu(II) by reductants. The values corresponding to reduction by O_2_^•−^ and the ascorbate anion (Asc^−^) are included for comparison purposes. The data correspond to aqueous solution, at pH = 7.4, and 298.15 K.
