# Supplementary material for: Capsaicin, a Powerful •OH-Inactivating Ligand
Source: Antioxidants (Basel). 2020 Dec 8;9(12):1247. doi: 10.3390/antiox9121247 (PMC7763808; doi:10.3390/antiox9121247)
Supplement: Supplementary file 1 [file antioxidants-09-01247-s001.pdf]

Capsaicin, a Powerful OH-Inactivating Ligand

Adriana Pérez-González,[1] Mario Prejano,[2] Nino Russo,[2] Tiziana Marino,[2*] Annia Galano[3*]

[1] *CONACYT - Universidad Autónoma Metropolitana - Iztapalapa. San Rafael Atlixco 186, Col. Vicentina. Iztapalapa. C. P. 09340. México D. F. México.*

[2] *Department of Chemistry and Chemical Technologies of Università della Calabria (UNICAL). Calabria, I-87036, Rende, ITALY.*

[3] *Departamento de Química. Universidad Autónoma Metropolitana-Iztapalapa. San Rafael Atlixco 186, Col. Vicentina. Iztapalapa. C. P. 09340. México D. F. México.*

Table of Contents

Figure S1. *RMSD trends calculated along molecular dynamics of neutral and anion forms of capsaicin molecule in water (top) and gas phase (bottom). On the right, the representative clusters extrapolated from the simulations. In blue, is represented the most populated clustered geometry.* 2

Figure S2. *Number of hydrogen bonds in the MD simulation of the neutral and anion form of the capsaicin.* 3

Figure S3. *Molar fractions of Capsaicin (CAP).* 4

Table S1. *Parameters adopted during MD simulations in AMBER package format* 5

Table S2. *Molar fraction of Capsaicin (8-methyl-N-vanillyl-6-nonenamide, CAP, pKa=10.1) at different pH.* 11

Table S3. *Acronyms and structures of the modeled complexes. The chelation routes (CR) in which of them is involved is specified, as well as the chelation sites (CS)_conformation (C) and structure.* 12

Table S4. *Gibbs free energies of reaction (ΔG°, kcal/mol), reorganization energies (λ, kcal/mol), Gibbs free energies of activation (ΔG≠, kcal/mol), rate coefficients (k_i^{app}, $M^{-1}s^{-1}$) and overall rate coefficients ($k_{overall}$, $M^{-1}s^{-1}$) for the reduction of the free Cu(II) by reductants. The values corresponding to reduction by $O_2^{\bullet -}$ and the ascorbate anion (Asc^-) are included for comparison purposes. The data correspond to aqueous solution, at pH=7.4, and 298.15 K.* 16

* E-mail: tiziana.marino65@unical.it
* E-mail: agal@xanum.uam.mx

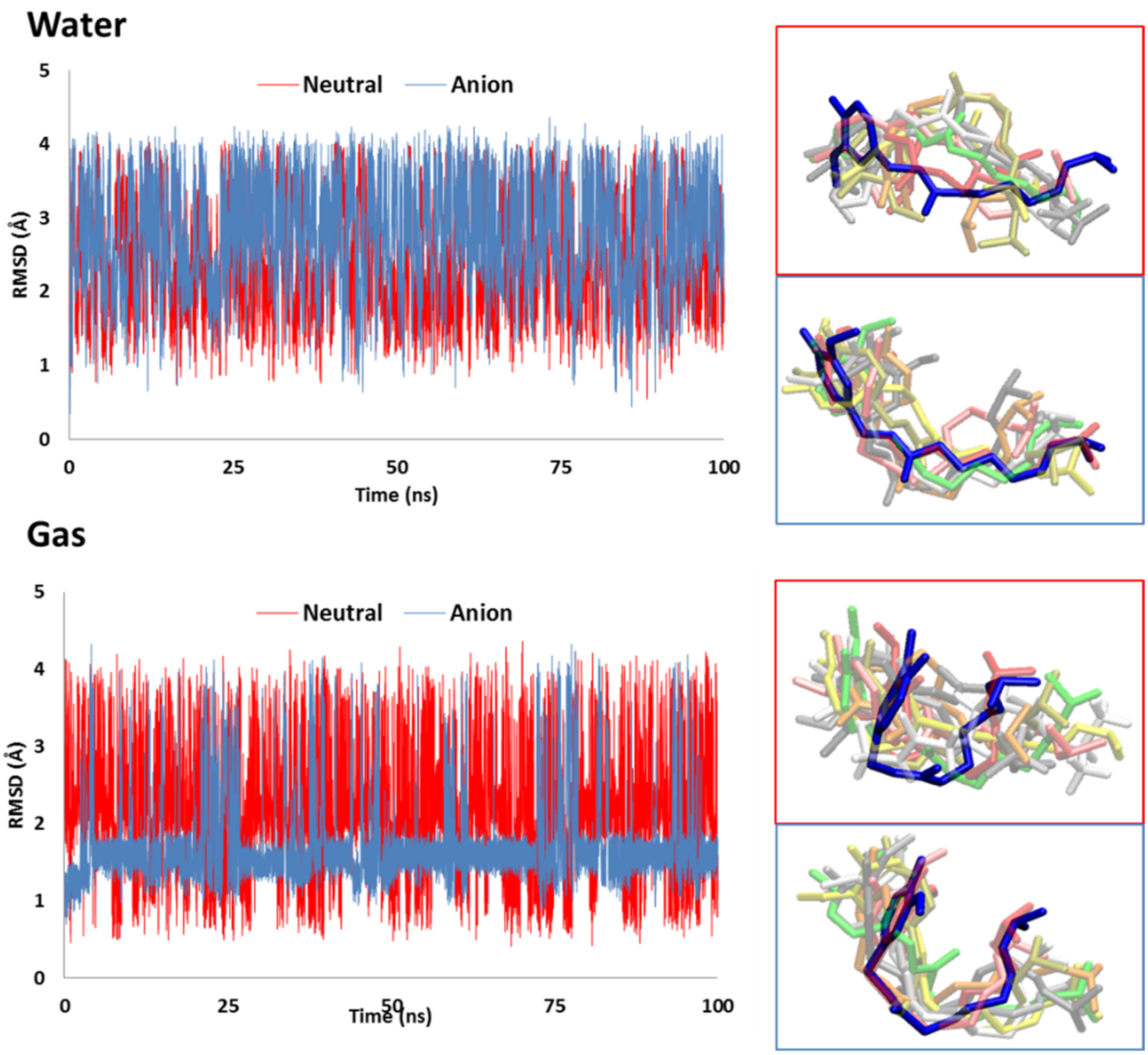

Figure S1. RMSD trends calculated along molecular dynamics of neutral and anion forms of capsaicin molecule in water (top) and gas phase (bottom). On the right, the representative clusters extrapolated from the simulations. In blue, is represented the most populated clustered geometry.

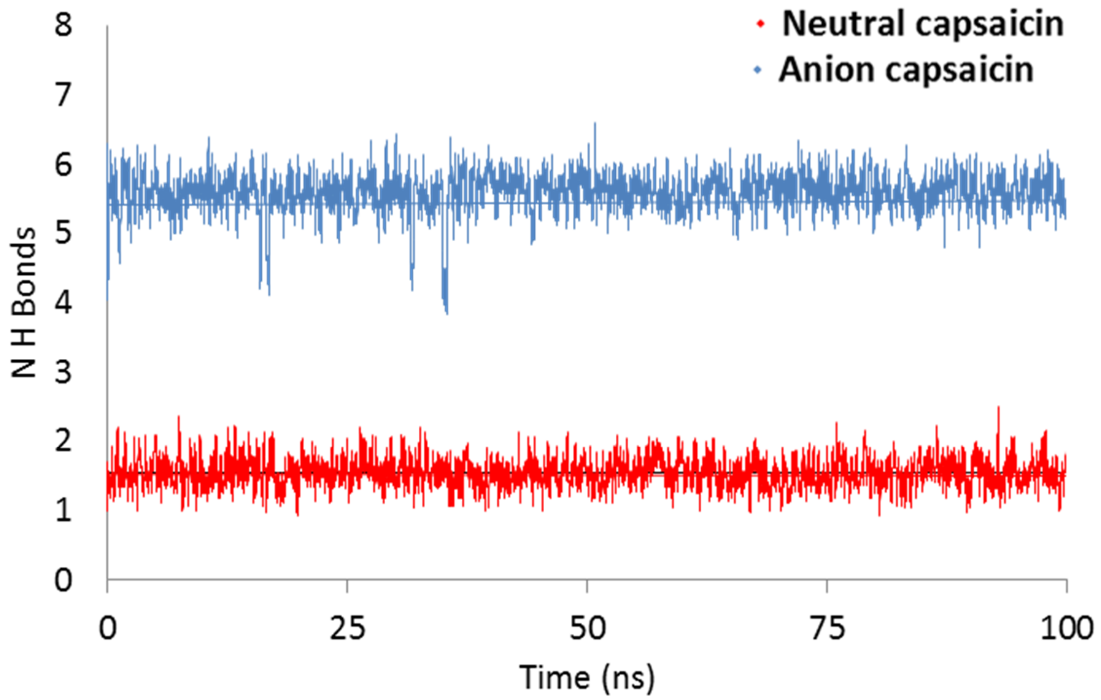

Figure S2. Number of hydrogen bonds in the MD simulation of the neutral and anion form of the capsaicin.

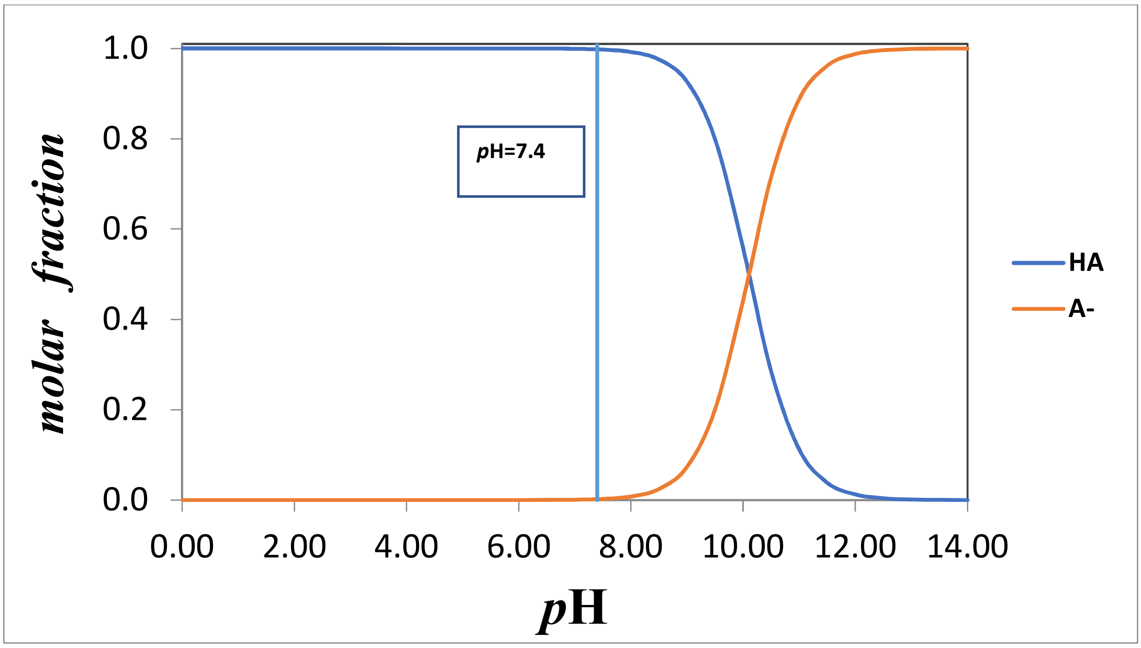

Figure S3. Molar fractions of Capsaicin (CAP).

Table S1. Parameters adopted during MD simulations in AMBER package format

Caps_n.prepc

0 0 2

This is a remark line
molecule.res
MOL XYZ 0
CHANGE OMIT DU BEG
0.0000

1 DUMM DU M 999.000 999.0 -999.0 .00000
2 DUMM DU M 999.000 -999.0 999.0 .00000
3 DUMM DU M -999.000 999.0 999.0 .00000
4 N1 n M -3.428000 0.711000 -0.554000 -0.617765
5 C7 c B -3.147000 1.509000 0.504000 0.661352
6 O1 o E -3.134000 1.102000 1.637000 -0.602586
7 C4 c3 3 -2.861000 2.963000 0.169000 -0.158299
8 C2 c3 3 -1.552000 3.177000 -0.609000 -0.015708
9 C1 c3 3 -0.310000 2.641000 0.106000 0.139548
10 C3 c3 3 0.983000 2.927000 -0.668000 -0.046821
11 C6 c2 B 2.204000 2.370000 0.015000 -0.187780
12 C8 c2 B 3.009000 1.458000 -0.497000 -0.329843
13 C5 c3 3 4.230000 0.883000 0.178000 0.394787
14 C11 c3 3 5.502000 1.243000 -0.601000 -0.399037
15 H15 hc E 6.385000 0.840000 -0.113000 0.094370
16 H16 hc E 5.621000 2.319000 -0.679000 0.094370
17 H17 hc E 5.468000 0.838000 -1.609000 0.094370
18 C12 c3 3 4.099000 -0.638000 0.333000 -0.399037
19 H18 hc E 4.019000 -1.122000 -0.637000 0.094370
20 H19 hc E 4.967000 -1.052000 0.838000 0.094370
21 H20 hc E 3.215000 -0.899000 0.905000 0.094370
22 H9 hc E 4.307000 1.321000 1.171000 -0.015908
23 H11 ha E 2.802000 1.072000 -1.486000 0.170748
24 H10 ha E 2.408000 2.756000 1.003000 0.121201
25 H5 hc E 0.907000 2.511000 -1.669000 0.036228
26 H6 hc E 1.094000 4.004000 -0.786000 0.036228
27 H1 hc E -0.247000 3.083000 1.099000 -0.004038
28 H2 hc E -0.401000 1.569000 0.252000 -0.004038
29 H3 hc E -1.623000 2.721000 -1.594000 -0.005116
30 H4 hc E -1.439000 4.244000 -0.783000 -0.005116
31 H7 hc E -3.693000 3.364000 -0.404000 0.054568
32 H8 hc E -2.825000 3.492000 1.113000 0.054568
33 H14 hn E -3.282000 1.077000 -1.467000 0.370229
34 C9 c3 M -3.585000 -0.730000 -0.433000 -0.035844
35 H12 h1 E -4.263000 -1.052000 -1.214000 0.108534
36 H13 h1 E -4.067000 -0.918000 0.515000 0.108534
37 C10 ca M -2.285000 -1.502000 -0.533000 0.043761
38 C14 ca B -1.914000 -2.137000 -1.699000 -0.246508
39 C16 ca S -0.701000 -2.817000 -1.788000 -0.272672
40 H23 ha E -0.407000 -3.314000 -2.694000 0.197403
41 H22 ha E -2.565000 -2.116000 -2.556000 0.173108
42 C13 ca M -1.426000 -1.559000 0.571000 -0.340153

43 H21 ha E -1.716000 -1.064000
1.477000 0.218164
44 C15 ca M -0.227000 -2.228000
0.486000 0.231506
45 O2 os S 0.681000 -2.349000
1.488000 -0.321975
46 C18 c3 3 0.421000 -1.748000
2.729000 0.044316
47 H25 h1 E 1.272000 -1.970000
3.356000 0.058439
48 H26 h1 E -0.475000 -2.158000
3.180000 0.058439
49 H27 h1 E 0.317000 -0.674000
2.629000 0.058439
50 C17 ca M 0.143000 -2.862000 -
0.706000 0.276596
51 O3 oh S 1.320000 -3.516000 -
0.794000 -0.583982
52 H24 ho E 1.771000 -3.451000
0.039000 0.409308

LOOP
C17 C16

IMPROPER
C9 C7 N1 H14
C4 N1 C7 O1
C8 C3 C6 H10
C6 C5 C8 H11
C9 C14 C10 C13
C10 C16 C14 H22
C14 C17 C16 H23
C10 C15 C13 H21
C13 C17 C15 O2
C16 C15 C17 O3

DONE
STOP

Caps_n.frcmod

remark goes here
MASS
n 14.010 0.530
c 12.010 0.616
o 16.000 0.434
c3 12.010 0.878
c2 12.010 0.360
hc 1.008 0.135
ha 1.008 0.135
hn 1.008 0.161
h1 1.008 0.135
ca 12.010 0.360
os 16.000 0.465
oh 16.000 0.465
ho 1.008 0.135

BOND
n -c 427.60 1.379
n -hn 403.20 1.013
n -c3 328.70 1.462
c -o 637.70 1.218
c -c3 313.00 1.524
c3-c3 300.90 1.538
c3-hc 330.60 1.097
c3-c2 326.80 1.510
c2-c2 569.40 1.334
c2-ha 343.10 1.088
c3-h1 330.60 1.097
c3-ca 321.00 1.516
ca-ca 461.10 1.398
ca-ha 345.80 1.086
ca-os 376.60 1.370
os-c3 308.60 1.432
ca-oh 384.00 1.364
oh-ho 371.40 0.973

ANGLE
n -c -o 74.220 123.050
n -c -c3 66.790 115.180
n -c3-h1 49.840 108.880
n -c3-ca 66.210 112.380
c -n -hn 48.330 117.550
c -n -c3 63.390 120.690
c -c3-c3 63.270 111.040
c -c3-hc 46.930 108.770
o -c -c3 67.400 123.200
c3-c3-c3 62.860 111.510
c3-c3-hc 46.340 109.800
c3-c3-c2 63.410 111.560
c3-c2-c2 64.060 123.630
c3-c2-ha 45.940 115.680
c2-c3-hc 46.990 110.360
c2-c2-ha 49.850 120.430
hc-c3-hc 39.400 107.580
hn-n -c3 45.800 117.680
c3-ca-ca 63.530 120.770
h1-c3-h1 39.240 108.460
h1-c3-ca 46.990 109.560
ca-ca-ca 66.620 120.020
ca-ca-ha 48.180 119.880
ca-ca-oh 69.520 119.900
ca-ca-os 69.580 119.200
ca-os-c3 62.520 117.960
os-c3-h1 50.800 109.780

```
ca-oh-ho   49.000    108.580

DIHE
n -c -c3-c3   1   0.100    0.000    -4.000
n -c -c3-c3   1   0.070    0.000     2.000
n -c -c3-hc   1   0.000    180.000   2.000
n -c3-ca-ca   1   0.000    0.000     2.000
c -n -c3-h1   1   0.000    0.000     2.000
c -n -c3-ca   1   0.000    0.000     2.000
c -c3-c3-c3   1   0.156    0.000     3.000
c -c3-c3-hc   1   0.156    0.000     3.000
o -c -n -hn   1   2.500    180.000  -2.000
o -c -n -hn   1   2.000    0.000     1.000
o -c -n -c3   1   2.500    180.000   2.000
o -c -c3-c3   1   0.000    180.000   2.000
o -c -c3-hc   1   0.800    0.000    -1.000
o -c -c3-hc   1   0.000    0.000    -2.000
o -c -c3-hc   1   0.080    180.000   3.000
c3-c -n -hn   1   2.500    180.000   2.000
c3-c -n -c3   1   0.000    0.000    -2.000
c3-c -n -c3   1   1.500    180.000   1.000
c3-c3-c3-c3   1   0.180    0.000    -3.000
c3-c3-c3-c3   1   0.250    180.000  -2.000
c3-c3-c3-c3   1   0.200    180.000   1.000
c3-c3-c3-hc   1   0.160    0.000     3.000
c3-c3-c3-c2   1   0.156    0.000     3.000
c3-c3-c2-c2   1   0.000    0.000     2.000
c3-c3-c2-ha   1   0.000    0.000     2.000
c3-c2-c2-c3   1   6.650    180.000  -2.000
c3-c2-c2-c3   1   1.900    180.000   1.000
c3-c2-c2-ha   1   6.650    180.000   2.000
c2-c3-c3-hc   1   0.156    0.000     3.000
c2-c2-c3-hc   1   0.380    180.000  -3.000
c2-c2-c3-hc   1   0.000    0.000    -2.000
c2-c2-c3-hc   1   1.150    0.000     1.000
hc-c3-c3-hc   1   0.150    0.000     3.000
hc-c3-c2-ha   1   0.000    0.000     2.000
ha-c2-c2-ha   1   6.650    180.000   2.000
hn-n -c3-h1   1   0.000    0.000     2.000
hn-n -c3-ca   1   0.000    0.000     2.000
c3-ca-ca-ca   1   3.625    180.000   2.000
c3-ca-ca-ha   1   3.625    180.000   2.000
h1-c3-ca-ca   1   0.000    0.000     2.000
ca-ca-ca-ha   1   3.625    180.000   2.000
ca-ca-ca-ca   1   3.625    180.000   2.000
ca-ca-ca-os   1   3.625    180.000   2.000
ca-ca-ca-oh   1   3.625    180.000   2.000
ca-ca-oh-ho   1   0.900    180.000   2.000
ha-ca-ca-ha   1   3.625    180.000   2.000
ha-ca-ca-oh   1   3.625    180.000   2.000
ca-ca-os-c3   1   0.900    180.000   2.000
ha-ca-ca-os   1   3.625    180.000   2.000
ca-os-c3-h1   1   0.383    0.000     3.000
os-ca-ca-oh   1   3.625    180.000   2.000

IMPROPER
c -c3-n -hn    1.1    180.0    2.0
c3-n -c -o     10.5   180.0    2.0     General improper torsional angle (2 general atom types)
c2-c3-c2-ha    1.1    180.0    2.0     Using default value
c3-ca-ca-ca    1.1    180.0    2.0
ca-ca-ca-ha    1.1    180.0    2.0     General improper torsional angle (2 general atom types)
ca-ca-ca-os    1.1    180.0    2.0     Using default value
ca-ca-ca-oh    1.1    180.0    2.0     Using default value

NONBON
  n      1.8240  0.1700
  c      1.9080  0.0860
  o      1.6612  0.2100
  c3     1.9080  0.1094
  c2     1.9080  0.0860
  hc     1.4870  0.0157
  ha     1.4590  0.0150
  hn     0.6000  0.0157
  h1     1.3870  0.0157
  ca     1.9080  0.0860
  os     1.6837  0.1700
  oh     1.7210  0.2104
  ho     0.0000  0.0000


Caps_a.prepc

   0   0   2

This is a remark line
molecule.res
MOL   XYZ  0
CHANGE    OMIT DU   BEG
  0.0000
   1  DUMM  DU   M     999.000    999.0    -999.0    .00000
   2  DUMM  DU   M     999.000   -999.0     999.0    .00000
   3  DUMM  DU   M    -999.000    999.0     999.0    .00000
   4  N1    n    M     -3.094000   -0.325000   -0.608000   -0.725022
   5  C7    c    B     -3.782000    0.192000    0.423000    0.821321
   6  O1    o    E     -4.404000   -0.472000    1.218000   -0.671813
```

7 C4 c3 3 -3.820000 1.717000 0.533000
-0.396859
8 C2 c3 3 -2.751000 2.536000 -0.198000
0.120609
9 C1 c3 3 -1.330000 2.385000 0.366000
-0.006457
10 C3 c3 3 -0.257000 2.889000 -
0.611000 -0.004386
11 C6 c2 B 1.138000 2.890000 -
0.036000 -0.304240
12 C8 c2 B 2.031000 1.937000 -
0.237000 -0.221025
13 C5 c3 3 3.428000 1.909000 0.335000
0.347738
14 C11 c3 3 4.445000 1.638000 -
0.782000 -0.396548
15 H15 hc E 5.460000 1.652000 -
0.390000 0.095546
16 H16 hc E 4.379000 2.388000 -
1.567000 0.095546
17 H17 hc E 4.264000 0.660000 -
1.217000 0.095546
18 C12 c3 3 3.550000 0.840000
1.430000 -0.396548
19 H18 hc E 3.354000 -0.146000
1.028000 0.095546
20 H19 hc E 4.554000 0.846000
1.852000 0.095546
21 H20 hc E 2.847000 1.033000
2.237000 0.095546
22 H9 hc E 3.636000 2.885000 0.773000
-0.052192
23 H11 ha E 1.768000 1.088000 -
0.850000 0.177728
24 H10 ha E 1.392000 3.730000
0.596000 0.130714
25 H5 hc E -0.277000 2.260000 -
1.496000 0.025547
26 H6 hc E -0.516000 3.896000 -
0.939000 0.025547
27 H1 hc E -1.263000 2.927000
1.307000 0.030681
28 H2 hc E -1.115000 1.347000
0.597000 0.030681
29 H3 hc E -2.759000 2.291000 -
1.258000 -0.035956
30 H4 hc E -3.037000 3.585000 -
0.150000 -0.035956
31 H7 hc E -4.808000 2.010000
0.185000 0.099493
32 H8 hc E -3.801000 1.933000
1.596000 0.099493
33 H14 hn E -2.495000 0.286000 -
1.112000 0.408570
34 C9 c3 M -2.730000 -1.744000 -
0.748000 0.194213
35 H12 h1 E -3.218000 -2.131000 -
1.638000 0.038215
36 H13 h1 E -3.150000 -2.257000
0.104000 0.038215
37 C10 ca M -1.238000 -1.911000 -
0.840000 -0.279649
38 C14 ca B -0.556000 -1.788000 -
2.036000 -0.124435
39 C16 ca S 0.829000 -1.773000 -
2.093000 -0.477824
40 H23 ha E 1.334000 -1.672000 -
3.038000 0.145448
41 H22 ha E -1.115000 -1.690000 -
2.957000 0.128965
42 C13 ca M -0.460000 -2.051000
0.329000 -0.242183
43 H21 ha E -0.976000 -2.158000
1.268000 0.169727
44 C15 ca M 0.906000 -2.057000
0.295000 0.147461
45 O2 os S 1.691000 -2.220000
1.404000 -0.360027
46 C18 c3 3 1.091000 -2.315000
2.646000 0.314502
47 H24 h1 E 1.888000 -2.408000
3.372000 -0.042984
48 H25 h1 E 0.444000 -3.187000
2.725000 -0.042984
49 H26 h1 E 0.502000 -1.431000
2.885000 -0.042984
50 C17 ca M 1.654000 -1.874000 -
0.939000 0.545193
51 O3 o E 2.898000 -1.807000 -
0.958000 -0.753261

LOOP
C17 C16

IMPROPER
C9 C7 N1 H14
C4 N1 C7 O1
C8 C3 C6 H10
C6 C5 C8 H11
C9 C14 C10 C13
C10 C16 C14 H22
C14 C17 C16 H23
C10 C15 C13 H21
C13 C17 C15 O2

```
 C16  C15  C17   O3

DONE
STOP

Caps_a.frcmod

remark goes here
MASS
n  14.010       0.530
c  12.010       0.616
o  16.000       0.434
c3 12.010       0.878
c2 12.010       0.360
hc 1.008        0.135
ha 1.008        0.135
hn 1.008        0.161
h1 1.008        0.135
ca 12.010       0.360
os 16.000       0.465

BOND
n -c   427.60   1.379
n -hn  403.20   1.013
n -c3  328.70   1.462
c -o   637.70   1.218
c -c3  313.00   1.524
c3-c3  300.90   1.538
c3-hc  330.60   1.097
c3-c2  326.80   1.510
c2-c2  569.40   1.334
c2-ha  343.10   1.088
c3-h1  330.60   1.097
c3-ca  321.00   1.516
ca-ca  461.10   1.398
ca-ha  345.80   1.086
ca-os  376.60   1.370
os-c3  308.60   1.432
ca-o   598.10   1.236

ANGLE
n -c -o    74.220     123.050
n -c -c3   66.790     115.180
n -c3-h1   49.840     108.880
n -c3-ca   66.210     112.380
c -n -hn   48.330     117.550
c -n -c3   63.390     120.690
c -c3-c3   63.270     111.040
c -c3-hc   46.930     108.770
o -c -c3   67.400     123.200
c3-c3-c3   62.860     111.510
c3-c3-hc   46.340     109.800
c3-c3-c2   63.410     111.560
c3-c2-c2   64.060     123.630
c3-c2-ha   45.940     115.680
c2-c3-hc   46.990     110.360
c2-c2-ha   49.850     120.430
hc-c3-hc   39.400     107.580
hn-n -c3   45.800     117.680
c3-ca-ca   63.530     120.770
h1-c3-h1   39.240     108.460
h1-c3-ca   46.990     109.560
ca-ca-ca   66.620     120.020
ca-ca-ha   48.180     119.880
ca-ca-o    71.370     123.260
ca-ca-os   69.580     119.200
ca-os-c3   62.520     117.960
os-c3-h1   50.800     109.780

DIHE
n -c -c3-c3   1    0.100        0.000        -4.000
n -c -c3-c3   1    0.070        0.000         2.000
n -c -c3-hc   1    0.000      180.000         2.000
n -c3-ca-ca   1    0.000        0.000         2.000
c -n -c3-h1   1    0.000        0.000         2.000
c -n -c3-ca   1    0.000        0.000         2.000
c -c3-c3-c3   1    0.156        0.000         3.000
c -c3-c3-hc   1    0.156        0.000         3.000
o -c -n -hn   1    2.500      180.000        -2.000
o -c -n -hn   1    2.000        0.000         1.000
o -c -n -c3   1    2.500      180.000         2.000
o -c -c3-c3   1    0.000      180.000         2.000
o -c -c3-hc   1    0.800        0.000        -1.000
o -c -c3-hc   1    0.000        0.000        -2.000
o -c -c3-hc   1    0.080      180.000         3.000
c3-c -n -hn   1    2.500      180.000         2.000
c3-c -n -c3   1    0.000        0.000        -2.000
c3-c -n -c3   1    1.500      180.000         1.000
c3-c3-c3-c3   1    0.180        0.000        -3.000
c3-c3-c3-c3   1    0.250      180.000        -2.000
c3-c3-c3-c3   1    0.200      180.000         1.000
c3-c3-c3-hc   1    0.160        0.000         3.000
c3-c3-c3-c2   1    0.156        0.000         3.000
c3-c3-c2-c2   1    0.000        0.000         2.000
c3-c3-c2-ha   1    0.000        0.000         2.000
c3-c2-c2-c3   1    6.650      180.000        -2.000
c3-c2-c2-c3   1    1.900      180.000         1.000
c3-c2-c2-ha   1    6.650      180.000         2.000
c2-c3-c3-hc   1    0.156        0.000         3.000
c2-c2-c3-hc   1    0.380      180.000        -3.000
c2-c2-c3-hc   1    0.000        0.000        -2.000
c2-c2-c3-hc   1    1.150        0.000         1.000
hc-c3-c3-hc   1    0.150        0.000         3.000
hc-c3-c2-ha   1    0.000        0.000         2.000
ha-c2-c2-ha   1    6.650      180.000         2.000
hn-n -c3-h1   1    0.000        0.000         2.000
```

hn-n -c3-ca 1 0.000 0.000 2.000
c3-ca-ca-ca 1 3.625 180.000 2.000
c3-ca-ca-ha 1 3.625 180.000 2.000
h1-c3-ca-ca 1 0.000 0.000 2.000
ca-ca-ca-ha 1 3.625 180.000 2.000
ca-ca-ca-ca 1 3.625 180.000 2.000
ca-ca-ca-os 1 3.625 180.000 2.000
ca-ca-ca-o 1 3.625 180.000 2.000
ha-ca-ca-ha 1 3.625 180.000 2.000
ha-ca-ca-o 1 3.625 180.000 2.000
ca-ca-os-c3 1 0.900 180.000 2.000
ha-ca-ca-os 1 3.625 180.000 2.000
ca-os-c3-h1 1 0.383 0.000 3.000
os-ca-ca-o 1 3.625 180.000 2.000

IMPROPER
c -c3-n -hn 1.1 180.0 2.0
c3-n -c -o 10.5 180.0 2.0 General improper torsional angle (2 general atom types)
c2-c3-c2-ha 1.1 180.0 2.0 Using default value
c3-ca-ca-ca 1.1 180.0 2.0
ca-ca-ca-ha 1.1 180.0 2.0 General improper torsional angle (2 general atom types)
ca-ca-ca-os 1.1 180.0 2.0 Using default value
ca-ca-ca-o 1.1 180.0 2.0 Using default value

NONBON
n 1.8240 0.1700
c 1.9080 0.0860
o 1.6612 0.2100
c3 1.9080 0.1094
c2 1.9080 0.0860
hc 1.4870 0.0157
ha 1.4590 0.0150
hn 0.6000 0.0157
h1 1.3870 0.0157
ca 1.9080 0.0860
os 1.6837 0.1700

Table S2. Molar fraction of Capsaicin (8-methyl-N-vanillyl-6-nonenamide, CAP, pKa=10.1) at different pH.

pH	[H+]	Neutral	Anion
0	1.00E+00	1.00E+00	7.94E-11
0.5	3.16E-01	1.00E+00	2.51E-10
1	1.00E-01	1.00E+00	7.94E-10
1.5	3.16E-02	1.00E+00	2.51E-09
2	1.00E-02	1.00E+00	7.94E-09
2.5	3.16E-03	1.00E+00	2.51E-08
3	1.00E-03	1.00E+00	7.94E-08
3.5	3.16E-04	1.00E+00	2.51E-07
4	1.00E-04	1.00E+00	7.94E-07
4.5	3.16E-05	1.00E+00	2.51E-06
5	1.00E-05	1.00E+00	7.94E-06
5.5	3.16E-06	1.00E+00	2.51E-05
6	1.00E-06	1.00E+00	7.94E-05
6.5	3.16E-07	1.00E+00	2.51E-04
7	1.00E-07	9.99E-01	7.94E-04
**7.4**	**3.98E-08**	**0.998**	**0.002**
8	1.00E-08	9.97E-01	2.51E-03
8.5	3.16E-09	9.92E-01	7.88E-03
9	1.00E-09	9.75E-01	2.45E-02
9.5	3.16E-10	9.26E-01	7.36E-02
10	1.00E-10	7.99E-01	2.01E-01
10.5	3.16E-11	5.57E-01	4.43E-01
10.7	2.00E-11	2.85E-01	7.15E-01
11	1.00E-11	1.12E-01	8.88E-01
11.5	3.16E-12	3.83E-02	9.62E-01
12	1.00E-12	1.24E-02	9.88E-01
12.5	3.16E-13	3.97E-03	9.96E-01
13	1.00E-13	1.26E-03	9.99E-01
13.5	3.16E-14	3.98E-04	1.00E+00
14	1.00E-14	1.26E-04	1.00E+00

Table S3. Acronyms and structures of the modeled complexes. The chelation routes (CR) in which of them is involved is specified, as well as the chelation sites (CS)_conformation (C) and structure.

CR	CS_C	Structure
IA	O1a	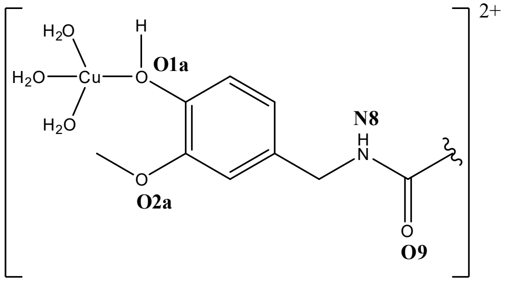
IA	O1a_ext	
IA	O2a	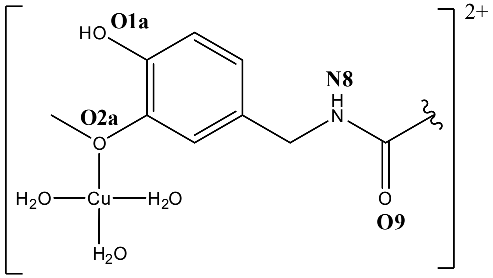
IA	O2a_ext	
IA	O9	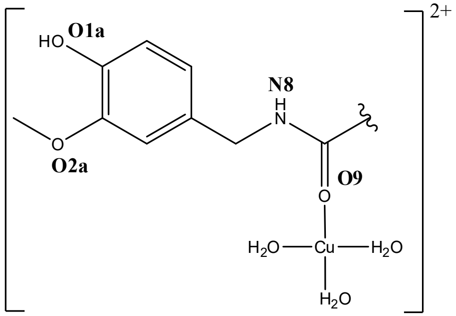
IA	O9_ext	

<table>
<tr><td>IB</td><td>O1a-O2a</td><td rowspan="2">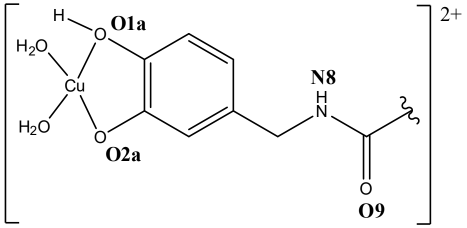</td></tr>
<tr><td>IB</td><td>O1a-O2a_ext</td></tr>
<tr><td>IIA
IIIA</td><td>O1a</td><td rowspan="2">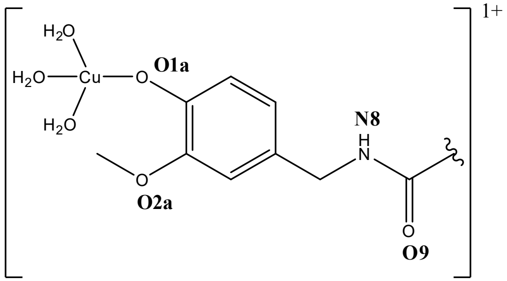</td></tr>
<tr><td>IIA
IIIA</td><td>O1a_ext</td></tr>
<tr><td>IIA</td><td>O2a_ext</td><td>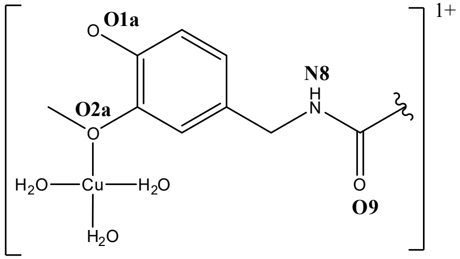</td></tr>
</table>

IIA	O9_ext	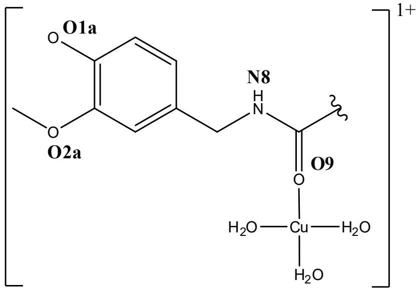
IIB<br>IIIB	O1a-O2a	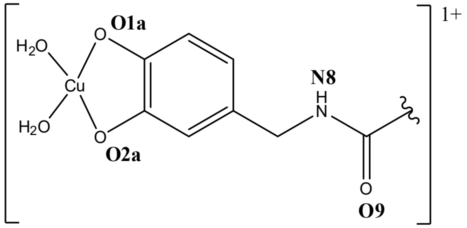
IIB<br>IIIB	O1a-O2a_ext	
IIIA	N8	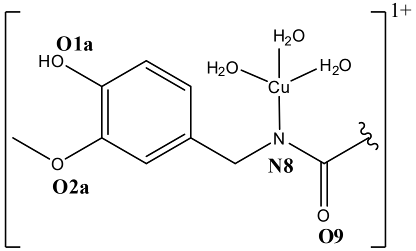
IIIA	N8_ext	
IIIB	N8-O9	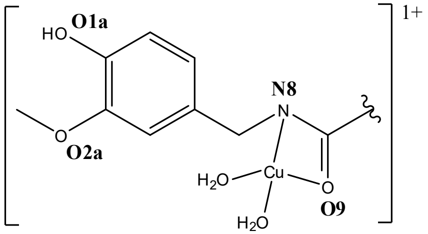

IVA	N8	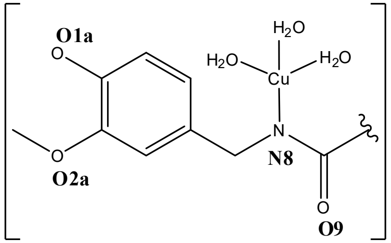
IVA	N8_ext	
IVB	N8-O9	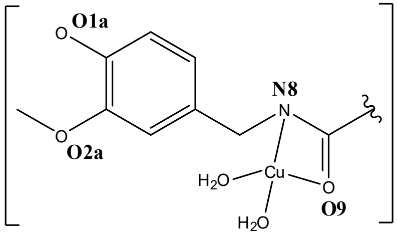

Table S4. Gibbs free energies of reaction ($\Delta G°$, kcal/mol), reorganization energies (λ, kcal/mol), Gibbs free energies of activation ($\Delta G^{\neq}$, kcal/mol), rate coefficients (k_i^{app}, $M^{-1}s^{-1}$) and overall rate coefficients ($k_{overall}$, $M^{-1}s^{-1}$) for the reduction of the free Cu(II) by reductants. The values corresponding to reduction by $O_2^{\bullet-}$ and the ascorbate anion (Asc^-) are included for comparison purposes. The data correspond to aqueous solution, at pH=7.4, and 298.15 K.

**Reductants**	**ΔG°**	**λ**	**ΔG≠**	k_i^{app}	$\frac{k_{O2\bullet-}^{app}}{k_i^{app}}$	$\frac{k_{Asc-}^{app}}{k_i^{app}}$
$O_2^{\bullet-}$	-24.01	51.87	3.74	4.46E+09		
Asc^-	-4.67	34.14	6.36	1.33E+08		
capN	5.07	32.01	10.74	7.39E+04	60342.80	1798.15
capN_ext	5.02	33.41	11.05	5.38E+03	829869.92	24729.16
capAn	-21.01	31.00	0.80	1.05E+07	424.49	12.65
capAn_ext	-20.21	31.28	0.98	4.96E+06	900.06	26.82
$k^{overall}$				1.55E+07	286.98	8.55
